# Supplementary material for: Dental Pulp Inflammation Initiates the Occurrence of Mast Cells Expressing the α1 and β1 Subunits of Soluble Guanylyl Cyclase
Source: Int J Mol Sci. 2023 Jan 4;24(2):901. doi: 10.3390/ijms24020901 (PMC9861465; doi:10.3390/ijms24020901)
Supplement: Supplementary file 1 [file ijms-24-00901-s001.zip › ijms-2049935-supplementary.pdf]

Supplement:

Dental Pulp Inflammation Initiates the Occurrence of Mast Cells Expressing α_1 and β_1 Subunits of Soluble Guanylyl Cyclase

Yüksel Korkmaz [1,*], Markus Plomann [2], Behrus Puladi [3], Aysegül Demirbas [4,5], Wilhelm Bloch [6] and James Deschner [1]

[1] Department of Periodontology and Operative Dentistry, University Medical Center, Johannes Gutenberg University Mainz, 55131 Mainz, Germany
[2] Center for Biochemistry, Faculty of Medicine, University of Cologne, 50931 Cologne, Germany
[3] Department of Oral and Maxillofacial Surgery, University Hospital RWTH Aachen, RWTH Aachen University, 52074 Aachen, Germany
[4] Department of Restorative Dentistry, Faculty of Dentistry, Mugla Sıtkı Kocman University, Mugla 48000, Turkey
[5] Department of Restorative Dentistry, Faculty of Dentistry, Ege University, Izmir 35040, Turkey
[6] Department of Molecular and Cellular Sport Medicine, German Sport University Cologne, 50933 Cologne, Germany
* Correspondence: yueksel.korkmaz@unimedizin-mainz.de

Characterization of the healthy and inflamed human dentin-pulp complex

The healthy dentin-pulp complex was observed in a structural (primary dentin, secondary dentin, predentin) and cellular (the odontoblast layer, the cell-free and cell-rich layers, the subodontoblastic plexus, nerve fibers and blood vessels) order (Figure S1A–D). In the carious dentin-pulp complex, deep dentin caries with local destruction of primary and secondary dentin was visible (Figure S1E). Reactive tertiary dentin was found beneath the carious lesion (Figure S1E,F). The odontoblast layer was found to be very thin (Figure S1F). In the area of the chronic and acute inflammatory areas, several neutrophilic granulocytes were found in the walls of blood vessels (Figure S1G,H).

In several cases, inflammation occurred in the dental pulp in mixed forms (necrotic changes, chronic, subacute and acute states of inflammation) (Figure S2A–D). MCT positive mast cells were found in the chronic and acute inflammatory areas mainly around the blood vessels (Figure S2E–H).

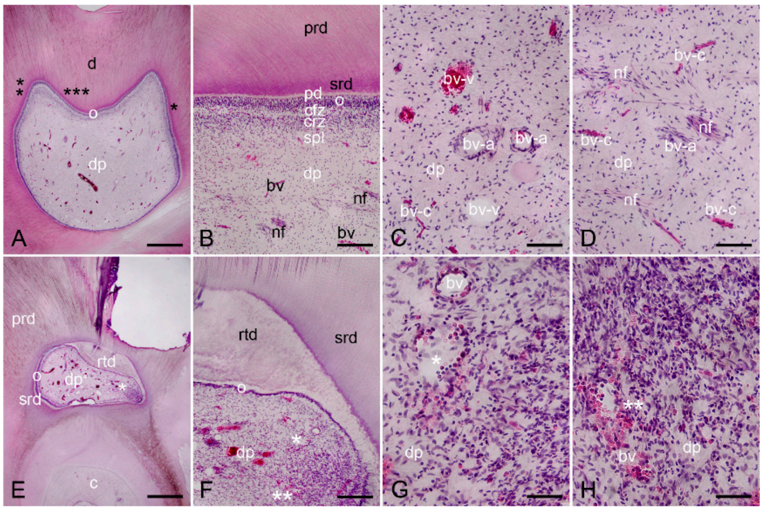

Figure S1. Histopathological characterization of healthy and inflamed human dentin-pulp complex by hematoxylin and eosin (HE) staining. The overview image of the dentin-pulp complex shows the structural order of dentin with primary dentin (prd), secondary dentin (srd), predentin (pd) and the structural order of the cell layers with odontoblast layer (o), cell-free zone (cfz), cell-rich zone (crz) and with numerous pulp cells in the human dental pulp (dp) (**A**,**B**). The arterial (bv-a) and venous (bv-v) blood vessels (bv) and nerve fibers (nf) are intact. Numerous pulp cells, some of which are closely associated with blood vessels and nerve fibers (**C**,**D**), are distributed throughout the dental pulp. In the chronic inflamed dental pulp, the primary dentin and secondary dentin are destroyed at the carious lesion areas, whereas, in response to caries, the reactive tertiary dentin (rtd) is formed (**E**). The carious lesion disrupts the structural order of the cell layers in the dental pulp (**F**). A thin layer of odontoblasts and mixed areas of inflammation with chronic and acute signs are visible beneath the carious lesion (**F**). In the chronic inflammatory region, a strong lymphocytic infiltrate with dilated blood vessels is seen, while in the transitional area from the chronic to the subacute inflammatory regions, numerous neutrophilic granulocytes and the other inflammatory cells leave the highly dilated blood vessels (* and ** in **F**). Blood vessels shown in (**F**) with * and ** are shown in detail in (**G**) with * and in (**H**) with **. Scale bars: (**A**,**E**) = 1 mm; (**B**,**F**) = 200 µm; (**C**,**D**,**G**,**H**) = 50 µm.

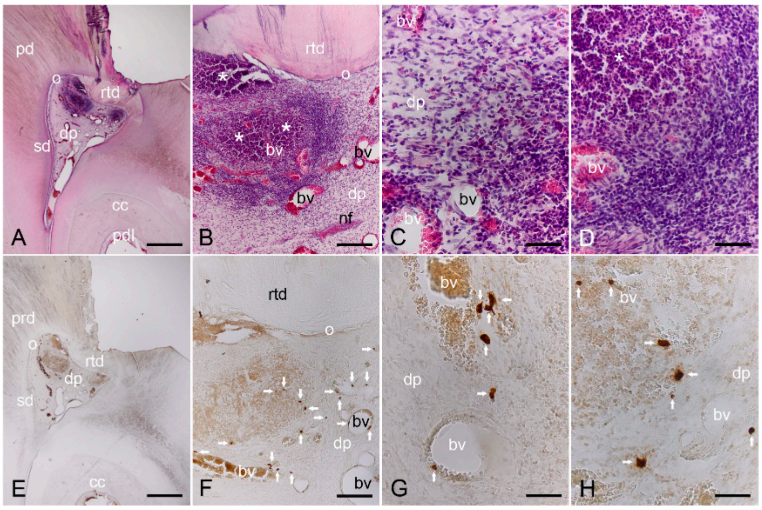

Figure S2. The characterization of the inflammation by hematoxylin and eosin (HE) staining and detection of MCT in mast cells in the inflamed human molar dental pulp (dp). In the chronic inflamed dentin–pulp complex, deep dentin carious lesion with destroyed primary dentin (prd), secondary dentin (srd), and with formation of reactionary tertiary dentin (rtd) areas are visible (**A**). Blood vessels (bv) are destroyed in the necrotic changed inflammatory regions (**B**). Beneath the carious lesion are necrotic altered inflammatory areas (asterisks), around which severe chronic and acute inflammatory regions with numerous different inflammatory cells and dilated blood vessels is seen in the overview image (**B**) and in the detailed images (**C**,**D**). In the consecutive section of the inflamed human dental pulp (**E**), MCT is identified in mast cells within chronic and acute inflamed areas which

are seen at overview (**F**) and detailed (**G**,**H**) images (arrows). Scale bars: (**A**,**E**) = 1 mm; (**B**,**F**) = 200 μm; (**C**,**D**,**G**,**H**) = 50 μm.

Detection of mast cell tryptase (MCT) in mast cells in the inflamed dental pulp

MCT was detected in mast cells of the chronic inflamed human dental pulp with deep dentin caries (Figure S3A,B).

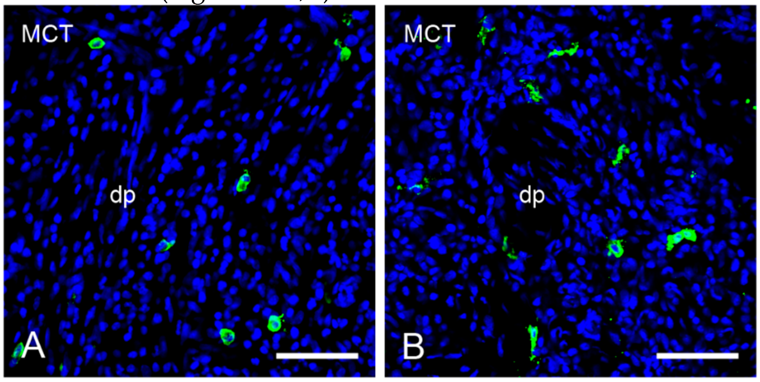

Figure S3. Detection of mast cells by the mast cell marker mast cell tryptase (MCT) in chronically inflamed human dental pulp (dp). Among the numerous round chronic inflammatory cells in the dental pulp, mast cells distinguished from other inflammatory cells by MCT (green) are clearly visible with larger cytoplasm and nuclei (**A**,**B**). Localization of DRAQ5 (blue) in the nuclei of numerous dental pulp immune cells indicates an inflammation in human dental pulp (**A**,**B**). Scale bars: (**A**,**B**) = 50 μm.

Expression of mast cell tryptase (MCT) with the α1- and β1-subunits of sGC in cells of the healthy dental pulp

In the cells of healthy human dental pulp, an immunoreactivity for MCT was not identified (Figure S4B,F). In healthy human dental pulp, the α1- and β1-subunits of sGC were detected in arterial and venous blood vessels (Figure S4C,D,G,H).

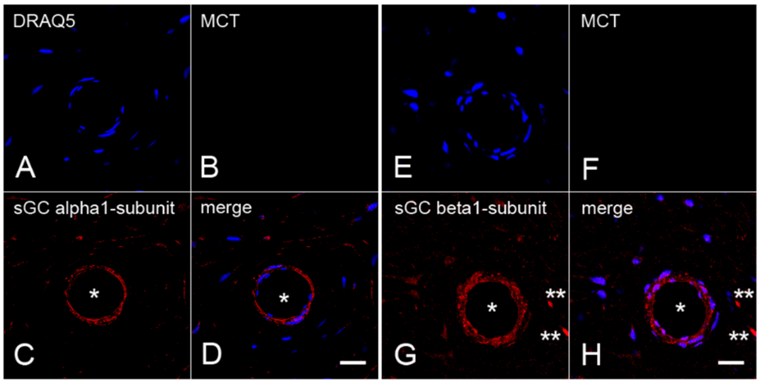

Figure S4. Colocalization of MCT with the α1- and β1-subunits of sGC in cells of healthy human dental pulp. The cells of the healthy dental pulp whose nuclei are stained with DRAQ5 (**A**,**D**,**E**,**H**) are negative for MCT (**B**,**D**,**F**,**H**). The α1- and β1-subunits of sGC are found in venous (asterisk; **C, D**) and arterial (asterisk; **G**,**H**) blood vessel walls. The β1-subunit of sGC is visible in capillaries (two asterisks; **G**,**H**) of the dental pulp. Scale bar = 20 μm.

Immunohistochemical controls

To test the specificities of the immunohistochemical reagents (normal goat serum: NGS, bovine serum albumin: BSA), the DAB substrate and the specificities of the secondary (goat anti-mouse IgG and goat anti-rabbit IgG) and primary antibodies, the primary antibodies were omitted from control incubations performed by the avidin-biotin-peroxidase complex method. In these control results, no immunoreactivity was observed in cells of the healthy (Figure S5A–D) and inflamed (Figure S5E–H) dental pulp.

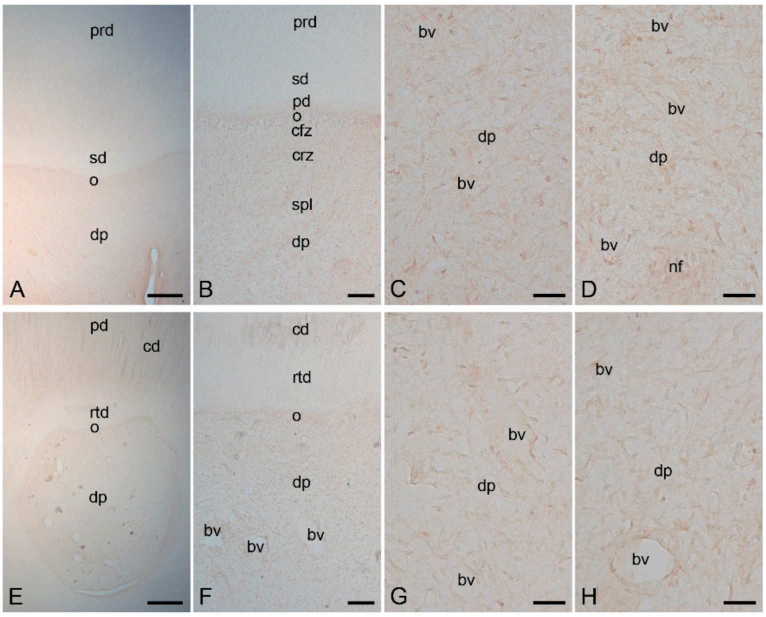

Figure S5. Controls of immunohistochemical reagents and secondary antibodies used for the avidin-biotin-peroxidase complex method. In the control incubations performed by omitting the primary antibodies, no specific immunostaining is seen in cells of the healthy (**A**–**D**) and inflamed (**E**–**H**) dental pulp (dp). In sections, only the normal background staining is visible through the DAB substrate. prd: primary dentin, srd: secondary dentin, pd: predentin, reactive tertiary dentin, o: the odontoblast layer (o), cfz: cell-free zone, crz: cell-rich zone, dp: dental pulp, bv: blood vessels, nf: nerve fibers. Scale bars: (**A**,**E**) = 500 µm, (**B**,**F**) = 100 µm, (**C**,**D**,**G**,**H**) = 30 µm.

To test the specificities of the immunohistochemical reagents, secondary and primary antibodies, the first and second primary antibodies were omitted from control incubations performed with the immunofluorescence double staining methods. In the control results of the double immunofluorescence method, no immunohistochemical reactivity was detected in the cells of the healthy (Figure S6A–D) and inflamed (Figure S6E–H) dentin-pulp complex, indicating the specificity of the immunohistochemical reagents and the primary and secondary antibodies that were used.

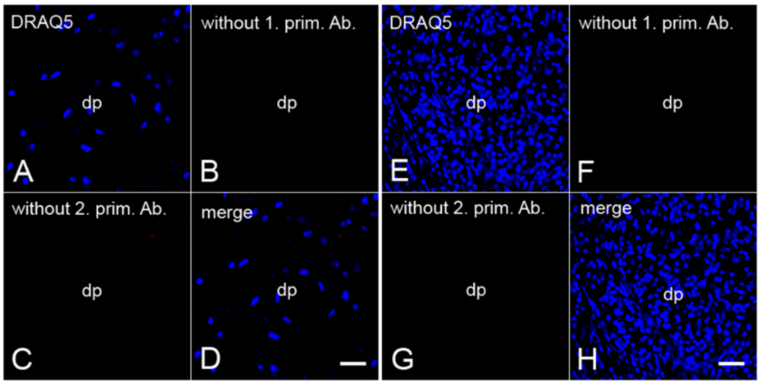

Figure S6. Controls of the double immunofluorescence method. In the immunofluorescence incubations, omission of first and secondary primary (prim.) antibodies (Ab.) was performed as control staining. No immunostaining is detected in cells of the healthy (**A**–**D**) and inflamed (**E**–**H**) dental pulp (dp). The detection of DRAQ5 (blue) in the nuclei of numerous immune cells of the dental pulp indicates severe inflammation in the human dental pulp (**E**,**H**). Scale bars: (**A**–**D**) = 20 µm, (**E**–**H**) = 50 µm.
